# (*E*)-4-Hy­droxy-2-{[(2-phenyl­eth­yl)iminium­yl]meth­yl}phenolate

**DOI:** 10.1107/S1600536812025408

**Published:** 2012-06-13

**Authors:** David Ortegon-Reyna, Cesar Garcias-Morales, Efren V. García-Báez, Armando Ariza-Castolo, Francisco J. Martínez-Martínez

**Affiliations:** a Facultad de Ciencias Químicas, Universidad de Colima, Carretera Coquimatlán-Colima, Coquimatlán Colima, México, CP 28400; b Laboratorio 26, Departamento de Química, Centro de Investigacion y de Estudios Avanzados del Instituto, Politécnico Nacional, Av. IPN 2508, San Pedro Zacatenco, CP 07360, México, D.F.; c Departamento de Ciencias-Básicas Químicas, Unidad Profesional Interdisciplinaria de Biotecnologia, del IPN, Avenida Acueducto s/n, Barrio la Laguna Ticoman, CP 07340, GAM, México, D.F.

## Abstract

The title Schiff base compound, C_15_H_15_NO_2_, crystallized as the iminium–phenolate zwitterion. The H atom is localized on the imine N atom, forming a strong intra­molecular hydrogen bond with the phenolate O atom, and giving rise to an *S*(6) ring motif. The mol­ecule has an *E* conformation about the C=N bond. In the crystal, mol­ecules are linked by O—H⋯O hydrogen bonds, forming chains propagating along [010]. There are also C—H⋯O inter­actions present.

## Related literature

For general background to the characteristics of Schiff bases, see: Krause *et al.* (1995 ▶); Hadjoudis *et al.* (2004 ▶). For related structures, see: Dominiak *et al.* (2006 ▶); Santos-Contreras *et al.* (2009 ▶); Ng (2008 ▶). For hydrogen-bond motifs, see: Bernstein *et al.* (1995 ▶).
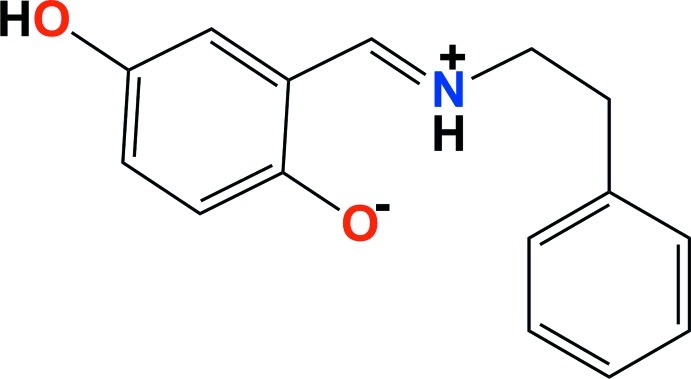


## Experimental

### 

#### Crystal data

C_15_H_15_NO_2_
*M* *_r_* = 241.28
Monoclinic, 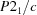

*a* = 9.5010 (19) Å
*b* = 12.936 (3) Å
*c* = 12.551 (4) Å
β = 124.81 (2)°
*V* = 1266.5 (6) Å^3^
*Z* = 4
Mo *K*α radiation
μ = 0.08 mm^−1^
*T* = 170 K
0.50 × 0.20 × 0.20 mm

#### Data collection

Nonius KappaCCD diffractometer
Absorption correction: for a sphere(Dwiggins, 1975 ▶) *T*_min_ = 0.861, *T*_max_ = 0.862
15718 measured reflections
2773 independent reflections
2367 reflections with *I* > 2σ(*I*)
*R*_int_ = 0.029

#### Refinement

*R*[*F* ^2^ > 2σ(*F* ^2^)] = 0.041
*wR*(*F* ^2^) = 0.107
*S* = 1.02
2773 reflections
163 parameters
H-atom parameters constrained
Δρ_max_ = 0.25 e Å^−3^
Δρ_min_ = −0.22 e Å^−3^

### 

Data collection: *COLLECT* (Nonius, 1998 ▶); cell refinement: *HKL* *SCALEPACK* (Otwinowski & Minor 1997 ▶); data reduction: *HKL* *DENZO*/*SCALEPACK* (Otwinowski & Minor, 1997 ▶); program(s) used to solve structure: *SIR2004* (Burla *et al.*, 2005 ▶); program(s) used to refine structure: *SHELXL97* (Sheldrick, 2008 ▶); molecular graphics: *ORTEP-3 for Windows* (Farrugia, 1997 ▶); software used to prepare material for publication: *WinGX* publication routines (Farrugia, 1999 ▶).

## Supplementary Material

Crystal structure: contains datablock(s) I, global. DOI: 10.1107/S1600536812025408/su2447sup1.cif

Structure factors: contains datablock(s) I. DOI: 10.1107/S1600536812025408/su2447Isup2.hkl

Additional supplementary materials:  crystallographic information; 3D view; checkCIF report

## Figures and Tables

**Table 1:** Hydrogen-bond geometry (Å, °)

*D*—H⋯*A*	*D*—H	H⋯*A*	*D*⋯*A*	*D*—H⋯*A*
N8—H8⋯O1	0.86	1.90	2.5884 (15)	137
O4—H4⋯O1^i^	0.82	1.87	2.6902 (15)	176
C6—H6⋯O4^ii^	0.95	2.59	3.2818 (18)	130

Symmetry codes: (i) 
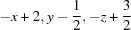
; (ii) 
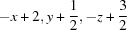
.

**Fig. 1.**
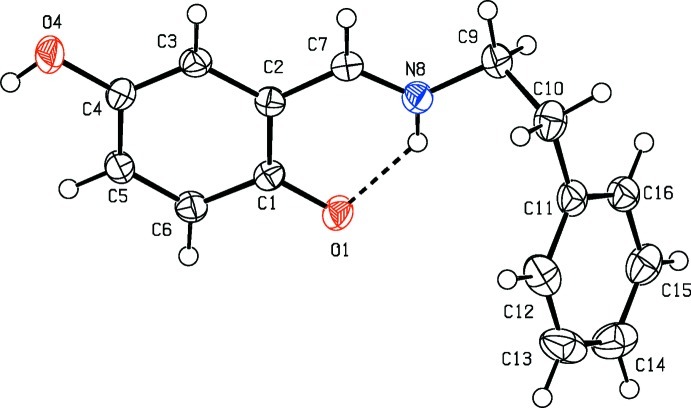
The molecular structure of the title compound with the atom labelling. Displacement ellipsoids are drawn at the 50% probability level. The intramolecular N-H···O bond is shown as a dashed line.

**Fig. 2.**
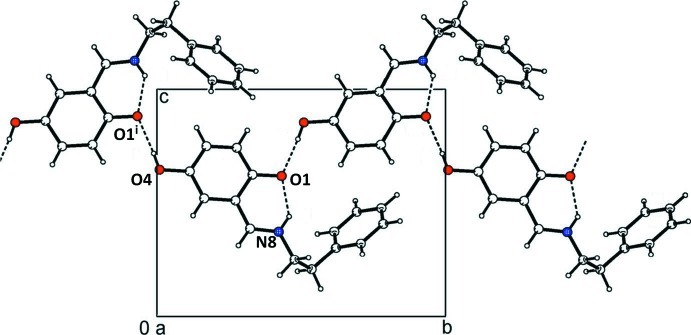
A partial view along the a axis of the crystal packing of the title compound. The N-H···O and O-H..O hydrogen bonds are shown as dashed lines [symmetry code: (i) -x+2, y-1/2, -z+3/2].

## supplementary crystallographic information

### Comment

Schiff base compounds are highly versatile compounds widely applied in different study fields. They have been used as selective prodrugs for histamine (Krause *et al.*, 1995). and as materials of interest because of their thermochromic and photochromic properties (Hadjoudis *et al.*, 2004). When an OH functionality is also present in the same molecule, tautomeric N—H or O—H equilibrium can be performed, thus these compounds can exist as O—H···N (imines), O···H—N (enamines) and also as N^+^—H···O^-^ zwitterionic forms (Dominiak *et al.*, 2006, Ng, 2008, Santos-Contreras *et al.*, 2009) Herein we demonstrate that the title compound exist as the N^+^—H···O^-^ zwitterionic form, with the H atom closing a six membered ring.

The title compound, Fig. 1, exists as a zwitterion with the hydrogen atom being localized on the imine N atom N8. The C1═O1 and N8═C7 bond lengths [1.3206 (14) and 1.2985 (16) Å, respectively] reveal significant double-bond character. The phenolate ring shows a certain distortion in the C—C bond lengths, but nevertheless an alternating long-short pattern is observed, in agreement with delocalized bonding. The C2—C7 bond distance of 1.4407 (16) Å is in the range for a single bond character. The intramolecular N8-H8···O1 hydrogen bond gives rise to the formation of an S(6) ring motif (Table 1; Bernstein *et al.*, 1995).

In the crystal, an O-H···O hydrogen bond links the molecules to form C(7) chains (Bernstein *et al.*, 1995) that propagate along the b axis direction (Table 1 and Fig. 2). There are also C-H···O interactions present (Table 1).

The title structure closely resembles that of *N*,-bis(2,5-dihydroxybezyl idene)-1,2-diaminobenzene (Dominiak *et al.*, 2006) and 4-chloro-2-[tris(hydroxymethyl)methyliminiomethyl]phenolate (Ng, 2008).

### Experimental

The title compound was prepared by condensation of 5-hydroxysalicylaldehyde (0.5 g, 3.62 mmol) with phenethylamine (0.438 g, 0.454 ml, 3.62 mmol) in toluene, at 298 K with stirring for 5 min. Orange crystals, suitable for X-ray analysis, were obtained by slow evaporation from a saturated dimethylsulfoxide solution [Yield of 0.78 g (90%); *M*.p. 396–397 K]. Spectroscopic data for the title compound are available in the archived CIF.

### Refinement

The amino and OH H atoms were located in a difference Fourier map. In the final cycles of refinement they and the C-bound H atoms were included in calculated positions and treated as riding atoms: O-H = 0.82 Å, N-H = 0.86 Å, C-H = 0.95 and 0.99 Å for CH and CH_2_ H atoms, respectively, with *U*_iso_(H) = k × Ueq(O,N,C) where k = 1.5 for the OH H atom and = 1.2 for other H atoms.

### Crystal data

**Table:**

C_15_H_15_NO_2_	*F*(000) = 512
*M**_r_* = 241.28	*D*_x_ = 1.265 Mg m^−^^3^
Monoclinic, *P*2_1_/*c*	Mo *K*α radiation, λ = 0.71073 Å
Hall symbol: -P 2ybc	Cell parameters from 14042 reflections
*a* = 9.5010 (19) Å	θ = 2.9–27.5°
*b* = 12.936 (3) Å	µ = 0.08 mm^−^^1^
*c* = 12.551 (4) Å	*T* = 170 K
β = 124.81 (2)°	Prism, orange
*V* = 1266.5 (6) Å^3^	0.50 × 0.20 × 0.20 mm
*Z* = 4	

### Data collection

**Table:**

Nonius KappaCCD diffractometer	2773 independent reflections
Radiation source: Enraf Nonius FR590	2367 reflections with *I* > 2σ(*I*)
Graphite monochromator	*R*_int_ = 0.029
Detector resolution: 9 pixels mm^-1^	θ_max_ = 27.5°, θ_min_ = 4.1°
CCD rotation images, thick slices scans	*h* = −10→12
Absorption correction: for a sphere (Dwiggins, 1975)	*k* = −16→16
*T*_min_ = 0.861, *T*_max_ = 0.862	*l* = −13→13
15718 measured reflections	

### Refinement

**Table:**

Refinement on *F*^2^	Primary atom site location: structure-invariant direct methods
Least-squares matrix: full	Secondary atom site location: difference Fourier map
*R*[*F*^2^ > 2σ(*F*^2^)] = 0.041	Hydrogen site location: inferred from neighbouring sites
*wR*(*F*^2^) = 0.107	H-atom parameters constrained
*S* = 1.02	*w* = 1/[σ^2^(*F*_o_^2^) + (0.0502*P*)^2^ + 0.3688*P*] where *P* = (*F*_o_^2^ + 2*F*_c_^2^)/3
2773 reflections	(Δ/σ)_max_ < 0.001
163 parameters	Δρ_max_ = 0.25 e Å^−^^3^
0 restraints	Δρ_min_ = −0.22 e Å^−^^3^

### Special details

**Table:**

Experimental. Spectroscopic data for the title compound: FT—IR by ATR (cm^-1^): 1643 (C═ N as intense band), 1496 (Asymmetrical C=C—O···H stretch), 3311 (free phenolic O—H intense broad band), 3059 (intramolecular hydrogen bonding N—H···O, as a weak broad band). LC/MS/TOF on HPLC-methanol solution, m/z (%) calculated: 242.1181 (100); found 242.1175 (100) [M+H]^+^, molecular formula C_15_H_15_NO_2_. ^1^H NMR (CDCl_3_, 300.1MHz): δ 6.83 (m, H5 and H6), 6.67 (s, H3), 8.12 (s, H7), 3.84 (t, H9), 3.00 (t, H10), 7.19–7.32 (m, H12—H16). ^13^C NMR (CDCl_3_, 75.4MHz): δ 147.5 (C1), 118.4 (C2), 116.6 (C3), 144.0 (C4), 119.9 (C5), 117.6 (C6), 164.5 (C7), 61.1 (C9), 37.3 (C10), 139.3 (C11), 128.9 (C13, C15), 128.5 (C12, C16), 126.4 (C14).
Geometry. Bond distances, angles *etc*. have been calculated using the rounded fractional coordinates. All su's are estimated from the variances of the (full) variance-covariance matrix. The cell e.s.d.'s are taken into account in the estimation of distances, angles and torsion angles
Refinement. Refinement of *F*^2^ against ALL reflections. The weighted *R*-factor *wR* and goodness of fit *S* are based on *F*^2^, conventional *R*-factors *R* are based on *F*, with *F* set to zero for negative *F*^2^. The threshold expression of *F*^2^ > σ(*F*^2^) is used only for calculating *R*-factors(gt) *etc*. and is not relevant to the choice of reflections for refinement. *R*-factors based on *F*^2^ are statistically about twice as large as those based on *F*, and *R*- factors based on ALL data will be even larger.

### Fractional atomic coordinates and isotropic or equivalent isotropic displacement parameters (Å^2^)

**Table:**

	*x*	*y*	*z*	*U*_iso_*/*U*_eq_	
O1	0.88467 (11)	0.43206 (6)	0.61806 (8)	0.0320 (3)	
O4	0.99566 (15)	0.00918 (7)	0.64559 (9)	0.0460 (3)	
N8	0.75925 (13)	0.42202 (8)	0.37321 (9)	0.0284 (3)	
C1	0.90916 (14)	0.33115 (8)	0.62390 (11)	0.0245 (3)	
C2	0.85954 (14)	0.27185 (8)	0.51050 (10)	0.0241 (3)	
C3	0.88858 (15)	0.16354 (9)	0.51983 (11)	0.0272 (3)	
C4	0.96636 (16)	0.11344 (9)	0.63837 (11)	0.0290 (3)	
C5	1.01496 (15)	0.17148 (9)	0.75028 (11)	0.0288 (3)	
C6	0.98611 (15)	0.27637 (9)	0.74326 (11)	0.0276 (3)	
C7	0.78312 (14)	0.32274 (9)	0.38709 (11)	0.0256 (3)	
C9	0.68816 (16)	0.47659 (9)	0.24949 (12)	0.0325 (4)	
C10	0.52322 (16)	0.53653 (10)	0.20739 (13)	0.0364 (4)	
C11	0.55332 (15)	0.62068 (9)	0.30222 (11)	0.0314 (3)	
C12	0.48187 (17)	0.61459 (12)	0.37397 (13)	0.0408 (4)	
C13	0.51032 (18)	0.69276 (14)	0.46060 (14)	0.0495 (5)	
C14	0.60966 (18)	0.77808 (13)	0.47683 (14)	0.0470 (5)	
C15	0.68349 (17)	0.78487 (10)	0.40787 (13)	0.0399 (4)	
C16	0.65511 (16)	0.70707 (10)	0.32130 (12)	0.0332 (4)	
H3	0.85445	0.12504	0.44421	0.0326*	
H4	1.03190	−0.01141	0.71879	0.0689*	
H5	1.06841	0.13740	0.83162	0.0345*	
H6	1.01819	0.31299	0.81963	0.0332*	
H7	0.74885	0.28207	0.31297	0.0307*	
H8	0.78653	0.45796	0.44009	0.0341*	
H9A	0.66198	0.42590	0.18137	0.0389*	
H9B	0.77472	0.52552	0.25890	0.0389*	
H10A	0.47519	0.56802	0.12123	0.0437*	
H10B	0.43734	0.48727	0.19847	0.0437*	
H12	0.41353	0.55663	0.36330	0.0490*	
H13	0.46162	0.68769	0.50873	0.0594*	
H14	0.62724	0.83176	0.53491	0.0565*	
H15	0.75308	0.84257	0.42000	0.0479*	
H16	0.70530	0.71235	0.27424	0.0398*	

### Atomic displacement parameters (Å^2^)

**Table:**

	*U*^11^	*U*^22^	*U*^33^	*U*^12^	*U*^13^	*U*^23^
O1	0.0424 (5)	0.0208 (4)	0.0295 (4)	0.0003 (3)	0.0185 (4)	−0.0023 (3)
O4	0.0827 (8)	0.0218 (4)	0.0385 (5)	0.0085 (5)	0.0376 (5)	0.0045 (4)
N8	0.0316 (5)	0.0272 (5)	0.0248 (5)	0.0016 (4)	0.0151 (4)	0.0004 (4)
C1	0.0234 (5)	0.0229 (5)	0.0270 (6)	−0.0020 (4)	0.0142 (5)	−0.0025 (4)
C2	0.0230 (5)	0.0248 (6)	0.0249 (6)	−0.0016 (4)	0.0140 (5)	−0.0007 (4)
C3	0.0324 (6)	0.0245 (6)	0.0272 (6)	−0.0024 (4)	0.0185 (5)	−0.0041 (4)
C4	0.0374 (6)	0.0209 (5)	0.0327 (6)	0.0000 (5)	0.0223 (5)	−0.0001 (4)
C5	0.0340 (6)	0.0269 (6)	0.0260 (6)	−0.0001 (5)	0.0175 (5)	0.0025 (4)
C6	0.0309 (6)	0.0266 (6)	0.0242 (6)	−0.0024 (5)	0.0150 (5)	−0.0034 (4)
C7	0.0250 (5)	0.0266 (6)	0.0262 (6)	−0.0006 (4)	0.0152 (5)	−0.0022 (4)
C9	0.0375 (7)	0.0311 (6)	0.0282 (6)	0.0038 (5)	0.0184 (5)	0.0049 (5)
C10	0.0318 (6)	0.0347 (7)	0.0327 (7)	0.0034 (5)	0.0125 (5)	0.0044 (5)
C11	0.0253 (6)	0.0348 (6)	0.0299 (6)	0.0077 (5)	0.0132 (5)	0.0087 (5)
C12	0.0288 (6)	0.0540 (8)	0.0385 (7)	0.0031 (6)	0.0185 (6)	0.0116 (6)
C13	0.0347 (7)	0.0833 (12)	0.0348 (7)	0.0142 (7)	0.0224 (6)	0.0074 (7)
C14	0.0340 (7)	0.0616 (10)	0.0349 (8)	0.0144 (7)	0.0134 (6)	−0.0054 (6)
C15	0.0324 (7)	0.0371 (7)	0.0403 (7)	0.0061 (5)	0.0149 (6)	0.0020 (5)
C16	0.0312 (6)	0.0356 (7)	0.0342 (7)	0.0058 (5)	0.0195 (5)	0.0065 (5)

### Geometric parameters (Å, º)

**Table:**

O1—C1	1.3206 (14)	C12—C13	1.394 (2)
O4—C4	1.3696 (16)	C13—C14	1.390 (3)
O4—H4	0.8200	C14—C15	1.394 (3)
N8—C7	1.2985 (16)	C15—C16	1.3898 (19)
N8—C9	1.4725 (16)	C3—H3	0.9500
N8—H8	0.8600	C5—H5	0.9500
C1—C6	1.4250 (17)	C6—H6	0.9500
C1—C2	1.4389 (16)	C7—H7	0.9500
C2—C7	1.4407 (16)	C9—H9A	0.9900
C2—C3	1.4199 (16)	C9—H9B	0.9900
C3—C4	1.3872 (17)	C10—H10A	0.9900
C4—C5	1.4175 (17)	C10—H10B	0.9900
C5—C6	1.3771 (17)	C12—H12	0.9500
C9—C10	1.546 (2)	C13—H13	0.9500
C10—C11	1.5148 (19)	C14—H14	0.9500
C11—C16	1.406 (2)	C15—H15	0.9500
C11—C12	1.406 (2)	C16—H16	0.9500
			
C4—O4—H4	109.00	C4—C3—H3	120.00
C7—N8—C9	123.74 (10)	C4—C5—H5	119.00
C9—N8—H8	118.00	C6—C5—H5	119.00
C7—N8—H8	118.00	C1—C6—H6	119.00
O1—C1—C2	121.62 (10)	C5—C6—H6	119.00
O1—C1—C6	121.22 (10)	N8—C7—H7	119.00
C2—C1—C6	117.17 (10)	C2—C7—H7	119.00
C1—C2—C7	119.95 (10)	N8—C9—H9A	109.00
C1—C2—C3	120.40 (10)	N8—C9—H9B	109.00
C3—C2—C7	119.64 (10)	C10—C9—H9A	109.00
C2—C3—C4	120.59 (11)	C10—C9—H9B	109.00
O4—C4—C3	119.65 (11)	H9A—C9—H9B	108.00
C3—C4—C5	119.21 (11)	C9—C10—H10A	109.00
O4—C4—C5	121.14 (10)	C9—C10—H10B	109.00
C4—C5—C6	121.21 (11)	C11—C10—H10A	109.00
C1—C6—C5	121.41 (11)	C11—C10—H10B	109.00
N8—C7—C2	122.58 (11)	H10A—C10—H10B	108.00
N8—C9—C10	111.47 (13)	C11—C12—H12	120.00
C9—C10—C11	113.07 (12)	C13—C12—H12	120.00
C10—C11—C12	121.30 (13)	C12—C13—H13	120.00
C10—C11—C16	120.53 (14)	C14—C13—H13	120.00
C12—C11—C16	118.18 (12)	C13—C14—H14	120.00
C11—C12—C13	120.64 (15)	C15—C14—H14	120.00
C12—C13—C14	120.24 (17)	C14—C15—H15	120.00
C13—C14—C15	119.97 (15)	C16—C15—H15	120.00
C14—C15—C16	119.87 (15)	C11—C16—H16	119.00
C11—C16—C15	121.08 (15)	C15—C16—H16	119.00
C2—C3—H3	120.00		
			
C9—N8—C7—C2	177.35 (15)	O4—C4—C5—C6	179.78 (16)
C7—N8—C9—C10	121.46 (15)	C3—C4—C5—C6	0.1 (3)
C6—C1—C2—C3	−0.5 (2)	C4—C5—C6—C1	−1.1 (2)
C6—C1—C2—C7	−179.18 (14)	N8—C9—C10—C11	62.54 (15)
O1—C1—C6—C5	−178.71 (15)	C9—C10—C11—C12	−113.64 (16)
C2—C1—C6—C5	1.3 (2)	C9—C10—C11—C16	65.94 (16)
O1—C1—C2—C7	0.8 (2)	C10—C11—C12—C13	−179.82 (14)
O1—C1—C2—C3	179.54 (14)	C16—C11—C12—C13	0.6 (2)
C7—C2—C3—C4	178.19 (15)	C10—C11—C16—C15	179.90 (13)
C1—C2—C3—C4	−0.5 (2)	C12—C11—C16—C15	−0.5 (2)
C3—C2—C7—N8	−177.33 (15)	C11—C12—C13—C14	0.2 (2)
C1—C2—C7—N8	1.4 (2)	C12—C13—C14—C15	−1.0 (2)
C2—C3—C4—C5	0.7 (2)	C13—C14—C15—C16	1.1 (2)
C2—C3—C4—O4	−178.96 (15)	C14—C15—C16—C11	−0.3 (2)

### Hydrogen-bond geometry (Å, º)

**Table:**

*D*—H···*A*	*D*—H	H···*A*	*D*···*A*	*D*—H···*A*
N8—H8···O1	0.86	1.90	2.5884 (15)	137
O4—H4···O1^i^	0.82	1.87	2.6902 (15)	176
C6—H6···O4^ii^	0.95	2.59	3.2818 (18)	130

Symmetry codes: (i) −*x*+2, *y*−1/2, −*z*+3/2; (ii) −*x*+2, *y*+1/2, −*z*+3/2.
